# Type B ankle fractures: a retrospective study of longer-term outcomes

**DOI:** 10.1186/s13104-017-2676-8

**Published:** 2017-07-28

**Authors:** Rajat Mittal, Prajith Jeyaprakash, Ian A. Harris, Justine M. Naylor

**Affiliations:** 1 Whitlam Orthopaedic Research Centre, Ingham Institute for Applied Medical Research, South Western Sydney Clinical School, UNSW, Sydney, Australia; 2 Orthopaedic Department, Liverpool Hospital, Locked Bag 7103, Liverpool BC, NSW 1871 Australia; 3 University of New South Wales, Sydney, Australia

**Keywords:** Ankle, Fracture, Patient-reported ankle function

## Abstract

**Objectives:**

Ankle fractures are common and can be treated with or without surgery. The aim of the present study was to compare patient reported outcomes between patients who sustained an Orthopaedic Trauma Association type 44-B1 ankle fracture who had either surgical or non-surgical fixation.

**Results:**

Forty-six people were recruited; 38 were treated non-surgically and 8 were treated surgically. Mean follow-up time was 24 and 25 months for surgical and non-surgical groups respectively. Baseline characteristics were similar between the two groups. On unadjusted analysis, there was no significant difference between the two groups with respect to any outcome. After adjusting for age and gender, the surgical group had a significantly lower outcome score with respect to the FAOQ. Surgical management was associated with a significantly lower patient-reported ankle function compared to non-surgical management for the treatment of patients with type 44-B1 ankle fracture after adjusting for age and gender. However, there was no significant difference between the two groups with respect to the general health outcomes or adverse events. Higher-level evidence is required to inform optimal practice for this common fracture.

## Introduction

Ankle fractures are common accounting for 9% of all adult fractures [1, 2]. The commonest type of ankle fracture is the Orthopaedic Trauma Association (OTA) classification 44-B1 ankle fracture (i.e. type B ankle fractures without substantial injury to the medial structures or talar shift) [3–7]. If the type B ankle fracture is combined with injury to the medial structures, as evident by a talar shift or medial malleolar fracture, surgical fixation is recommended, as it is considered unstable. The 44-B1 ankle fracture is considered stable as the medial structures are intact, however, despite this, there has been uncertainty in the treatment of it. This decision of whether the 44-B1 ankle fracture should be treated with surgery or not is often left to the judgement of the treating orthopaedic surgeon and consequently, shows considerable practice variation [8].

Advocates for surgical management for the 44-B1 ankle fracture emphasise the importance of achieving an anatomic reduction with internal fixation thereby limiting the potential for displacement and instability [4, 9, 10]. Advocates for non-surgical management argue that surgery is more expensive and is associated with more adverse events (refs—pre this study), and believe non-surgical management is associated with good long term. Certainly, at the time this study commenced, no prospective study had been published demonstrating that either approach was superior to the other for this type of fracture [10–14].

In the absence of longer-term data comparing surgical to non-surgical management of 44-B1 ankle fractures, this study was conducted to determine whether those treated surgically and who had a minimum 12 month follow-up had better patient-reported ankle function, quality of life and less adverse events.

## Main text

### Methods

This was a retrospective study of people with a 44-B1 ankle fracture and who were more than 1 year post presentation at the hospital involved. All those who presented at one of two teaching hospitals (Liverpool or St. George Hospitals in Sydney, NSW, Australia) between March 2008 to July 2009 and who required an ankle x-ray were retrospectively identified. People between 18 and 65 years and who had a type 44-B1 ankle fracture with minimal talar shift identified radiographically, were eligible to participate. People were excluded if they had talar shift (significant talar shift was defined as medial clear space being at least 2 mm wider than the superior clear space on mortise x-ray view of the ankle); were pregnant, or an open fracture at the time of presentation. Those eligible were contacted by mail and invited to participate in a survey capturing their longer-term outcomes. Those who did not respond after 2 weeks were contacted by telephone. Contact details were found using the hospital database, contacting the local general practitioner or next of kin.

Outcomes were collected over the telephone from those consenting to participate. The primary outcomes were the American Academy of Orthopaedic Surgeons Foot and Ankle Outcomes Questionnaire (FAOQ) and the physical component score (PCS) of the general health survey short form 12v2 (SF-12v2). Both these surveys are validated outcome measures used previously in a trauma setting [3, 5, 15–17]. Secondary outcomes were the mental component score (MCS) of the SF-12v2 and adverse events. Adverse events were unplanned/repeat surgery; infection; deep vein thrombosis or pulmonary embolus; death; neurological injury.

The sample size for the study was dictated by the number of eligible people who had presented in the study time-frame. The study time-frame was dictated by the online availability of ankle radiographs. Ideally, a minimum 130 people with 65 in each treatment group would provide 80% power to detect a 0.5 SD difference between the primary outcomes with 95% confidence if there was one. The Students’ t test was used to compare normally distributed continuous variables while the Mann–Whitney-U was used for continuous variables that were not normally distributed. The Chi square test was used for categorical variables. A significance level of p < 0.05 was considered significant. Statistical analysis was conducted using SAS 9.4 (Cary, NC, USA). Multiple linear regression was conducted for the primary outcomes adjusting for age and gender.

### Results

**Fig. 1:**
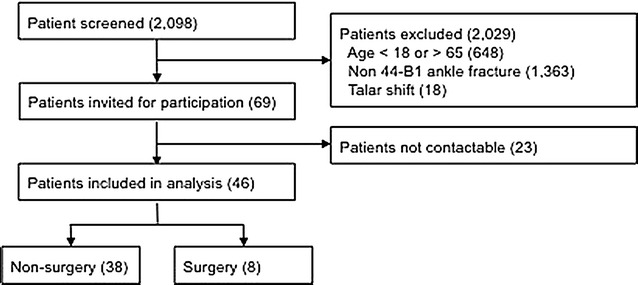
Cohort ascertainment flowchart

A total of 2098 patients who presented to St. George and Sutherland Hospitals between March 2008 and July 2009 had ankle x-rays. Of these, 69 patients were eligible to participate. Twenty-three patients were not contactable, but all who were agreed to participate in the study. Thus, a total of 46 patients were recruited with 38 managed non-surgically in a cast and 8 managed surgically. The cohort ascertainment flowchart is shown in Fig. 1. The mean follow-up time was 24 months.

Baseline participant characteristics were similar between the two groups (Table 1). The baseline characteristics of non-respondents were similar to those who responded (Table 1).

**Table 1:** Baseline demographics of surgical vs. non-surgical groups and respondents vs. non-respondents

Variable	No-surgery (n = 38)	Surgery (n = 8)	p value
Age, mean (sd)	43 (14)	39 (17)	0.51
Males, no. (%)	21 (57%)	2 (25%)	0.14

Variable	Respondents (n = 46)	Non-respondents (n = 23)	p value
Age, mean (sd)	43 (14.7)	41 (17.1)	0.62
Males, no. (%)	24 (52%)	12 (52%)	1.0

Unadjusted analyses demonstrated that there was no significant difference between the non-surgical and surgical groups with respect to the FAOQ (mean difference 7.1, favouring the non-surgical group; 95% CI −2.4 to 16.6; p = 0.12), the PCS (mean difference 3.7, favouring the non-surgical group; 95% CI −2.8 to 10.3) or the MCS (mean difference 6.0, favouring the non-surgical group; 95% CI −1.4 to 13.5). There was also no significant difference in the proportion of patients who reported adverse events (3% in the non-surgical group vs. 25% in the surgical group; p = 0.07) and the number reporting adverse events was small. A participant in the non-surgical group had a skin infection that settled after a course of oral antibiotics. One participant in the surgical group had a deep vein thrombosis after the operation and another required ongoing treatment for their ankle at the time of follow-up. None of the participants required late surgery. A summary of the results is shown in Table 2.

**Table 2:** Unadjusted results comparing non-surgery vs. surgery

Variable	Non-surgical (n = 38)	Surgical (n = 8)	Mean difference or odds ratio (95% CI)	p value
FAOQ, mean (sd)^a^	50.3 (6.3)	43.2 (11.3)	7.1 (−2.4 to 16.6)	0.12
PCS, mean (sd)^a^	50.8 (8.0)	47.1 (9.7)	3.7 (−2.8 to 10.3)	0.26
MCS, mean (sd)^a^	54.2 (9.4)	48.1 (9.7)	6.0 (−1.4 to 13.5)	0.11
Any adverse event, no. (%)^b^	1 (3%)	2 (25%)	12.3 (1.0 to 158)	0.07

^a^Difference is mean difference (95% CI)

^b^Difference is odds ratio (95% CI)

Multiple linear regression, adjusting for age and gender, showed that surgery was associated with a significantly lower outcome score with respect to FAOQ (Table 3). Although surgery was associated with lower scores with respect to PCS and MCS, these were not statistically, significantly different.

**Table 3:** Effect of surgery on outcome scores after adjusting for age and gender

	Estimate of effect (95% CI)	p value
Effect of surgery on FAOQ	−6.14 (−11.7 to −0.6)	0.03
Effect of surgery on PCS	−3.2 (−8.8 to 2.4)	0.25
Effect of surgery on MCS	−5.7 (−13.2 to 1.9)	0.14

### Discussion

This was a retrospective study of adult patients aged between 18 and 65 with an isolated type 44-B1 ankle fracture with minimal talar shift and at least 12 months follow-up. We found that surgical management was associated with a significantly lower ankle function score after adjusting for age and gender. There was no significant difference between surgical and non-surgical management with respect to the health related quality of life or adverse events. Given the smaller than expected sample size, the study was theoretically underpowered to detect significant differences in any outcomes. Thus, the lack of difference between the groups in quality of life or adverse events, despite the significantly lower ankle function scores, may reflect a lack of statistical power.

Despite our small sample size and the retrospective nature of our study, our findings accord with related studies published recently, after our study commenced. A recent systematic review conducted by Donken et al. showed there was insufficient evidence to justify surgical management of type B ankle fractures [10]. This was because the prevailing RCTs identified by the review included patients with either different patterns of ankle fractures and/or with significant talar shift that potentially confounds the need for surgery [12, 18–22]. Long-term observational studies of 44-B1 ankle fractures also published after our study have revealed good results for surgical or non-surgical management [13, 23, 24]. Van Shcie-Can der Weert et al. conducted a retrospective study similar to the present study [25]. In their study of 124 patients with type 44-B1 ankle fractures 59% with treated non-surgically while 41% were treated surgically. Both groups had good clinical outcomes.

### Strengths

That all patients who were contactable participated and that those who were not contactable had similar baseline characteristics to those who participated, lends confidence to the notion that our findings are representative.

### Conclusion

In this retrospective cohort, surgical management was associated with a significantly lower ankle function when compared with non-surgical management for the treatment of patients with type 44-B1 ankle fracture with minimal talar shift after adjusting for gender and age. Randomised controlled trials are required to confirm the findings of this study and also account for possible confounders which may contribute to differences in longer-term patient-reported outcomes between the surgical and non-surgical approaches.

## Limitations

Our main limitations here are the small sample and the retrospective design, the latter preventing our ability to confirm similar ankle function and quality of life between the two groups immediately prior to injury. Thus, it is possible, pre-morbid differences in ankle function may have contributed to the observed differences in ankle function more than 1 year after injury. Further, we are unable to comment on the decision-making process that led to a patient being managed surgically or not. Evidence of clinical instability may have influenced the management pathway and thus may confound the longer-term results. Finally, differences in return to physical or occupational activity or physiotherapy management may contribute to the observed differences in longer-term ankle function between the two groups.

## Abbreviations

OTA: Orthopaedic Trauma Association
FAOQ: American Academy of Orthopaedic Surgeons Foot and Ankle Outcomes Questionnaire
PCS: physical component score
SF-12v2: general health survey short form 12v2
MCS: mental component score

## References

[1] Dindo D, Demartines N, Clavien P-A (2004). Classification of surgical complications: a new proposal with evaluation in a cohort of 6336 patients and results of a survey. Ann Surg.

[2] Court-Brown CM, Caesar B (2006). Epidemiology of adult fractures: a review. Injury.

[3] Ware JJ, Kosinski M, Keller SD (1996). A 12-item short-form health survey: construction of scales and preliminary tests of reliability and validity. Med Care.

[4] Michelson JD (1995). Fractures about the ankle. J Bone Joint Surg Am.

[5] Ware JE, Kosinski M, Turner-Bowker DM, Gandek B (2002). User’s manual for the SF-12v2 health survey with a supplement documenting SF-12 health survey.

[6] Burwell HN, Charnley AD (1965). The treatment of displaced fractures at the ankle by rigid internal fixation and early joint movement. J Bone Joint Surg Br.

[7] Resnick B, Nahm ES (2001). Reliability and validity testing of the revised 12-item short-form health survey in older adults. J Nurs Meas.

[8] Ansari U, Adie S, Harris IA, Naylor JM (2011). Practice variation in common fracture presentations: a survey of orthopaedic surgeons. Injury.

[9] Michelson JD (2003). Ankle fractures resulting from rotational injuries. J Am Acad Orthop Surg.

[10] Donken Christian CMA, Al-Khateeb H, Verhofstad Michael HJ, van Laarhoven Cornelis JHM (2012). Surgical versus conservative interventions for treating ankle fractures in adults. Cochrane Database Syst Rev.

[11] Bauer M, Bengner U, Johnell O, Redlund-Johnell I (1987). Supination-eversion fractures of the ankle joint: changes in incidence over 30 years. Foot Ankle.

[12] Bauer M, Bergstrom B, Hemborg A, Sandegard J (1985). Malleolar fractures: nonoperative versus operative treatment. A controlled study. Clin Orthop.

[13] Bauer M, Jonsson K, Nilsson B (1985). Thirty-year follow-up of ankle fractures. Acta Orthop Scand.

[14] Sanders DW, Tieszer C, Corbett B (2012). Society OBOTCOT. Operative versus nonoperative treatment of unstable lateral malleolar fractures: a randomized multicenter trial. J Orthop Trauma.

[15] Kiely JM, Brasel KJ, Guse CE, Weigelt JA (2006). Correlation of SF-12 and SF-36 in a trauma population. J Surg Res.

[16] Johanson NA, Liang MH, Daltroy L, Rudicel S, Richmond J, Johanson NA (2004). American Academy of Orthopaedic Surgeons lower limb outcomes assessment instruments. Reliability, validity, and sensitivity to change. J Bone Joint Surg Am.

[17] So S, Harris IA, Naylor JM, Adie S, Mittal R (2011). Correlation between metal allergy and treatment outcomes after ankle fracture fixation. J Orthop Surg.

[18] Phillips WA, Schwartz HS, Keller CS, Woodward HR, Rudd WS, Spiegel PG (1985). A prospective, randomized study of the management of severe ankle fractures. J Bone Joint Surg Am.

[19] Pakarinen HJ, Flinkkila TE, Ohtonen PP, Hyvonen PH, Lakovaara MT, Leppilahti JI (2011). Syndesmotic fixation in supination-external rotation ankle fractures: a prospective randomized study. Foot Ankle Int.

[20] Kortekangas TH, Pakarinen HJ, Savola O, Niinimaki J, Lepojarvi S, Ohtonen P (2014). Syndesmotic fixation in supination-external rotation ankle fractures: a prospective randomized study. Foot Ankle Int.

[21] Makwana NK, Bhowal B, Harper WM, Hui AW (2001). Conservative versus operative treatment for displaced ankle fractures in patients over 55 years of age. A prospective, randomised study. J Bone Joint Surg Br.

[22] Rowley DI, Norris SH, Duckworth T (1986). A prospective trial comparing operative and manipulative treatment of ankle fractures. J Bone Joint Surg Br.

[23] Kristensen KD, Hansen T (1985). Closed treatment of ankle fractures. Stage II supination-eversion fractures followed for 20 years. Acta Orthop Scand.

[24] Stufkens SAS, van den Bekerom MPJ, Kerkhoffs GMMJ, Hintermann B, van Dijk CN (2011). Long-term outcome after 1822 operatively treated ankle fractures: a systematic review of the literature. Injury.

[25] Van Schie-Van der Weert EM, Van Lieshout EMM, De Vries MR, Van der Elst M, Schepers T (2012). Determinants of outcome in operatively and non-operatively treated Weber-B ankle fractures. Arch Orthop Trauma Surg.

